# Intravitreal injections with a low consumption technique have a low infection rate

**DOI:** 10.1038/s41433-023-02753-z

**Published:** 2023-09-27

**Authors:** Jesse Gale, Sarah H. Welch, Rachael Niederer

**Affiliations:** 1 Surgery & Anaesthesia, University of Otago Wellington, Wellington, New Zealand; 2 Ophthalmology, Te Whatu Ora Te Toka Tumai Auckland, Auckland, New Zealand; 3 Ophthalmology, University of Auckland, Auckland, New Zealand

**Keywords:** Outcomes research, Drug therapy

As intravitreal injections (IVI) have become the most common ophthalmic procedure, streamlined care pathways are required to deliver large numbers of IVI safely, effectively and comfortably. Ophthalmologists use a range of IVI techniques, with some using more personal protective equipment, antiseptics and antibiotics, injected and topical anaesthesia, and disposable sterile equipment for the injection (a high-consumption technique), while others use very few single use items (a relatively lower-consumption technique). The high volume of IVI means that this consumption of single use sterile supplies and medications is an important consideration for both healthcare costs and environmental impact [1, 2]. Ophthalmologists need evidence to support their decisions on IVI practice: it is important to demonstrate the safety of low-consumption practices to allow savings to be enjoyed by all [3, 4].

We sought to measure the rate of post-IVI endophthalmitis for a standardised relatively low-consumption technique (in use since 2018), and compare it to published rates in the same city between 2007 and 2014, when ophthalmologists injected using a variety of non-standardised techniques [5]. In both time periods, the predominant medication was bevacizumab, prepared into single use syringes by compounding pharmacies. As Auckland, New Zealand has one very centralised service for ocular emergencies, and prospective tracking of endophthalmitis, it provided an ideal opportunity to capture every case and trace the origin. The Department of Ophthalmology, Te Whatu Ora Te Toka Tumai Auckland (previously Auckland District Health Board) has maintained a prospective database of all endophthalmitis cases since 2016. All patients who underwent intravitreal or aqueous sampling were notified to one of the authors (RN) for investigation and inclusion in the database as appropriate. This was cross-checked by ward staff emailing RN for endophthalmitis cases, and a yearly review of all sampling to ensure no missed cases. This real-time monitoring allowed early identification of cluster endophthalmitis, as well as monitoring of local disease prevalence and microorganisms.

In Auckland, a nurse injector system has delivered over 95% of the public hospital IVI in recent years, a total over 17,000 per year in 2022, for a city of around 1.6 million. The nurse injectors followed the following protocol, which standardised the infection control measures and use of equipment. In a clean procedure room, the injector wore sterile gloves and a mask, no other personal protective equipment was used. Topical amethocaine anaesthesia alone was used, unless a patient requested injected subconjunctival lignocaine 1% (drawn from a vial that lasted the whole day). Sterile minims were used, one per patient, but after this study period, minims were replaced with multi-use bottles of amethocaine. A customised sterile pack with paper wrapping provided a sterile field containing five squares of gauze, five cotton tip sticks, a single use plastic galley pot for ~20 mL iodine 5% (chlorhexidine used when iodine contact allergy), and a single use plastic calliper. Added to this sterile field for the procedure were the needle, prefilled sterile syringe, a reused sterile metal speculum, 5 mL saline and lubricant eye drop (both in single use plastic containers) (see Fig. 1). Patients were asked not to talk, to look down and away, speculum was placed and the supero-temporal injection site is marked and iodine applied to the injection site with cotton tip stick before injection.

**Fig. 1:**
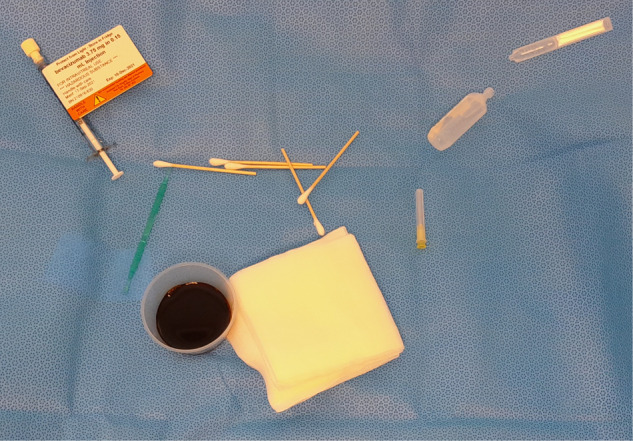
Photograph of the standardised setup for IVI in Auckland from 2018. Note that a reusable speculum and sterile gloves are not shown. In 2022, the use of single use callipers was discontinued.

In the period 1 January 2018 to 30 June 2022 a total of 67,696 IVI were given in the Auckland public system, and 15 cases of IVI endophthalmitis were recorded (1/4513 or 0.022%, 95% confidence interval, CI, 0.011–0.035%). This rate was exactly the same when considering only bevacizumab IVI (11/49,907 or 0.022%, CI 0.011–0.034%). This compared to a rate of IVI endophthalmitis of 21/39,657 (1/1888 or 0.053%, CI 0.033–0.081%) in the published study of Auckland ophthalmologists’ bevacizumab IVI [5]. Using a Poisson regression method to compare these rates the incidence rate ratio (IRR) = 0.477, *p* = 0.062, indicating no statistically significant difference in infection rate (this was also true for only bevacizumab IVI, IRR = 0.473, *p* = 0.080).

For the 15 endophthalmitis cases, the median time to presentation following intravitreal injection was 6.5 days (interquartile range, IQR 3–9 days) and median presenting corrected acuity was 6/60 (IQR 6/21–count fingers). Six cases (40.0%) were culture positive, and only one was due to oral flora (*Streptococcus mitis*). Initial treatment was vitreous tap and intravitreal injection of antibiotics (tap/inject) for 11 eyes and primary vitrectomy in 4 eyes. For those undergoing primary tap/inject, six had subsequent vitrectomy, of which 5 were performed within 24 h of presentation with endophthalmitis. The median final visual acuity was 6/15 (IQR 6/9–6/120), with severe vision loss occurring in 4 eyes (26.7%).

This simple report highlights that a nurse injector system can result in standardised technique with lower consumption of single use sterile supplies, and maintain very low infection rates. This supports ophthalmologists in making changes to their practice to minimise consumption without compromising the safety or quality of care.

## Data Availability

We are happy to share data with any reasonable request. The underlying data have not been made available publicly.
